# Antibiotic Resistance and Virulence Phenotypes of Recent Bacterial Strains Isolated from Urinary Tract Infections in Elderly Patients with Prostatic Disease

**DOI:** 10.3390/pathogens6020022

**Published:** 2017-05-31

**Authors:** Cristina Delcaru, Paulina Podgoreanu, Ionela Alexandru, Nela Popescu, Luminiţa Măruţescu, Coralia Bleotu, George Dan Mogoşanu, Mariana Carmen Chifiriuc, Marinela Gluck, Veronica Lazăr

**Affiliations:** 1 Earth, Environmental and Life Sciences Section, Research Institute of the University of Bucharest (ICUB), 91–95 Independenţei Avenue, 0500088 Bucharest, Romania; cristina.delcaru@lpiancului.ro (C.D.); carmen_balotescu@yahoo.com (M.C.C.); veronica.lazar2009@gmail.com (V.L.); 2 Iancului Private Laboratory, 060101 Bucharest, Romania; podgoreanu.paula@yahoo.com (P.P.); nicoleta.popescu24@gmail.com (N.P.); 3 Department of Microbiology & Immunology, Faculty of Biology, University of Bucharest, 1–3 Portocalelor Lane, Sector 6, 060101 Bucharest, Romania; lumidascalu@yahoo.com; 4 Ştefan S. Nicolau Institute of Virology, 285 Mihai Bravu Avenue, 030304 Bucharest, Romania; c_bleotu@yahoo.com; 5 Department of Pharmacognosy & Phytotherapy, Faculty of Pharmacy, University of Medicine and Pharmacy of Craiova, 2 Petru Rareş Street, 200349 Craiova, Romania; 6 Dr. Gluck Private Practice, 060101 Bucharest, Romania; contact@gluckmed.ro

**Keywords:** urinary tract infections, antibiotic resistance, biofilm

## Abstract

Acute bacterial prostatitis is one of the frequent complications of urinary tract infection (UTI). From the approximately 10% of men having prostatitis, 7% experience a bacterial prostatitis. The purpose of this study was to investigate the prevalence of uropathogens associated with UTIs in older patients with benign prostatic hyperplasia and to assess their susceptibility to commonly prescribed antibiotics as well as the relationships between microbial virulence and resistance features. Uropathogenic *Escherichia coli* was found to be the most frequent bacterial strain isolated from patients with benign prostatic hyperplasia, followed by *Enterococcus* spp., *Enterobacter* spp., *Klebsiella* spp., *Proteus* spp., *Pseudomonas aeruginosa*, and *Serratia marcescens*. Increased resistance rates to tetracyclines, quinolones, and sulfonamides were registered. Besides their resistance profiles, the uropathogenic isolates produced various virulence factors with possible implications in the pathogenesis process. The great majority of the uropathogenic isolates revealed a high capacity to adhere to HEp-2 cell monolayer in vitro, mostly exhibiting a localized adherence pattern. Differences in the repertoire of soluble virulence factors that can affect bacterial growth and persistence within the urinary tract were detected. The Gram-negative strains produced pore-forming toxins—such as hemolysins, lecithinases, and lipases—proteases, siderophore-like molecules resulted from the esculin hydrolysis and amylases, while *Enterococcus* sp. strains were positive only for caseinase and esculin hydrolase. Our study demonstrates that necessity of investigating the etiology and local resistance patterns of uropathogenic organisms, which is crucial for determining appropriate empirical antibiotic treatment in elderly patients with UTI, while establishing correlations between resistance and virulence profiles could provide valuable input about the clinical evolution and recurrence rates of UTI.

## 1. Introduction

Urinary tract infections (UTIs) are among the most common indications for antibiotic use in the community and in health services [1]. UTIs associated with catheter use account for 30–40% of all nosocomial infections and are the most common source of Gram-negative bacteremia in hospitalized patients [2]. Treatment of this type of prostatitis consists of long-term antimicrobial therapy and often the symptoms reoccur shortly after the acute phase due to the inability of antimicrobial agents to penetrate prostate tissue and achieve optimal concentrations to eradicate the infection. The antibiotics commonly used to treat prostatitis are fluoroquinolones, which show the highest rate of infection eradication, macrolides, tetracyclines, and trimethoprim [3]. Enterococci have become an increasingly common cause of UTI, representing more than 30% of all bacterial isolates. Enterococci are intrinsically resistant to many antimicrobials and can also develop resistance to a large range of antibiotics [4]. The presence of enterococci in the urinary tract is often asymptomatic [5]. Unfortunately, broad-spectrum antibiotics are increasingly used for UTIs, which is a risk factor for the development of strains resistant to vancomycin (VRE) and VRE colonization [6]. In 2015, Romania was described at a European level as one of the countries with the largest consumption of narrow spectrum antibiotics (first generation cephalosporins, erythromycin, penicillin V), but also those with a broad spectrum (third-generation cephalosporins and IV-penicillins associated with β-lactamase inhibitors). It has been estimated that 600,000 of Romanian citizens consume antibiotics irresponsibly [7]. According to the latest official reports issued by the European Center for Disease Prevention and Control (ECDC) for 2015, Romania was third among countries with invasive infections produced by *E. coli* strains with higher resistance to fluoroquinolones (30.7%) followed by third-generation cephalosporins (26.8%) and aminoglycosides (18.4%) and the first with combined resistance (to three or more antimicrobial groups, among which piperacillin-tazobactam, ceftazidime, fluoroquinolones, aminoglycosides, and carbapenems) [8]. Prostate enlargement, also called benign prostatic hyperplasia (BPH), represents an important risk factor for urinary tract infections (UTIs) and bacterial prostatitis in men [9,10]. This structural abnormality is mainly associated with aging and most often affects men who are 60 years of age and older [11]. This chronic condition can prevent the bladder from emptying completely, which increases the likelihood that bacteria will grow and trigger an infection [10]. UTIs are among the most common indications for antibiotic use in the community and health services [12]. There is a paucity of evidence-based guidelines for the management of UTI specifically in the older men population [13]. Studies suggest that UTI is incorrectly diagnosed in as many as 40% of hospitalized older people [14]. The increasing prevalence of health care associated infection and emerging antibiotic resistance highlights the importance of obtaining a firm diagnosis and appropriate antibiotic treatment, as well as avoiding the use of broad-spectrum antibiotics [15]. The knowledge of the resistance profiles of uorpathogenic microorganisms involved in acute/chornic prostatitis will help to the improvement of the antimicrobial therapy and thus, to the decrease of the costs of hospitalization and treatment. This will reduce the duration of treatment and assure a faster recovery of the patient’s health status, limiting antibiotic resistance dissemination in hospitals and in the community. Here, we investigated the prevalence of uropathogens associated with UTIs in older patients with benign prostatic hyperplasia and assessed the susceptibility of these pathogens to commonly prescribed antibiotics. The relationships between microbial virulence and resistance determinants were also evaluated [16].

## 2. Results

### 2.1. Prevalence of Uropathogenic Bacteria Associated with Urinary Tract Infections in Older Patients with Benign Prostatic Hyperplasia

A group of 85 outpatients diagnosed with benign prostatic hyperplasia with recurrent UTI was included in this study. Urinalysis resulted in 70% positive cultures with significant bacteriuria (i.e., >10^5^ colony forming units—CFU/mL). In the positive specimens, *Escherichia coli* was the most predominant isolated microorganism (60%), followed by other *Enterobacteriaceae*, of which *Klebsiella* spp. (8.2%), *Proteus* spp. (7%), *Enterobacter* spp. (5%), *Serratia marcescens* (1.1%), and *Morganella morganii* (1.1%). *Enterococcus* spp. were detected in 15.3% and *Streptococcus agalactiae* in 2.3% of the urine specimens.

### 2.2. Antimicrobial Susceptibility

**Figure 1:**
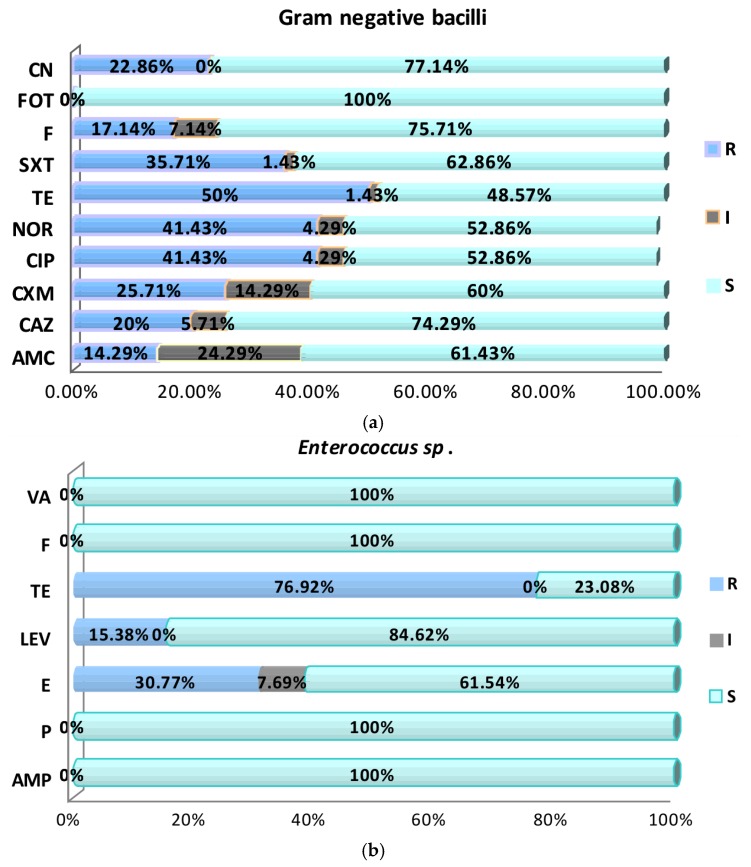
Graphic representation of the antibiotic susceptibility profiles in the Gram-negative (**a**) and *Enterococcus* sp. (**b**) analyzed uropathogenic strains. CN: Gentamicin; FOT: Fosfomycin; F: Nitrofurantoin; SXT: Trimethoprim–Sulfamethoxazole; TE: Tetracycline; NOR: Norfloxacin; CIP: Ciprofloxacin; CXM: Cefuroxime; CAZ: Ceftazidime; AMC: Amoxicillin–Clavulanic acid; VA: Vancomycin; LEV: Levofloxacin; E: Erythromycin; P: Penicillin; AMP: Ampicillin; R: Resistant; I: Intermediary; S: Sensitive.

**Figure 2:**
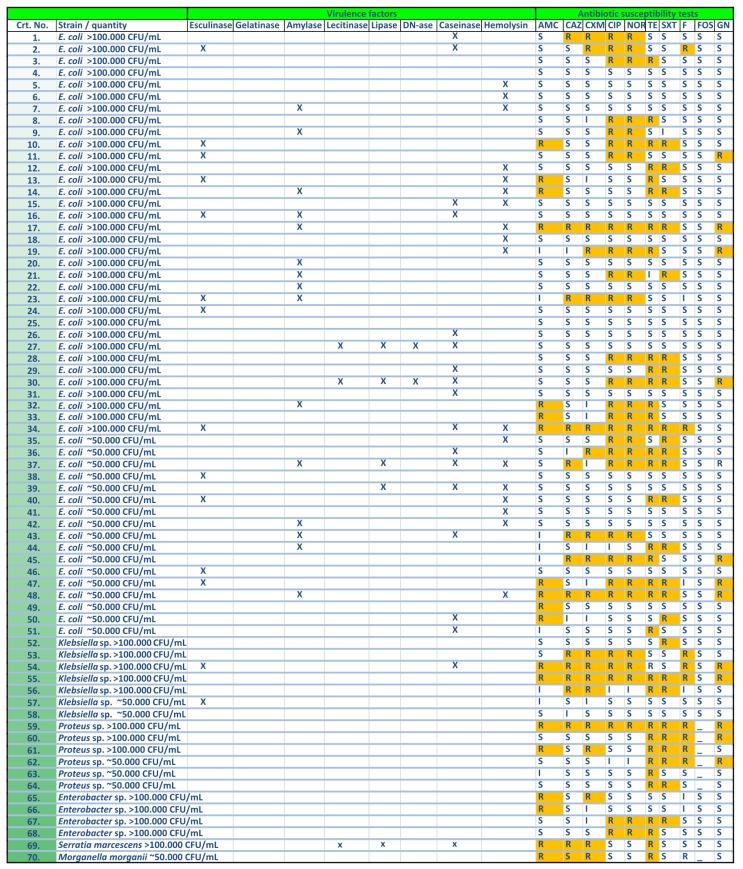
Representation of the individual profiles of soluble virulence factors and antibiotic resistance profiles in the analyzed Gram-negative strains.

The enterobacterial strains exhibited high antibiotic susceptibility rates to fosfomycin (100%), gentamicin (77.14%), nitrofurantoin (75.71%), ceftazidime (74.29%), sulfamethoxazole (62.86%), amoxicillin–clavulanic acid (61.43%), cefuroxime (60%), fluoroquinolones (52.86%), and tetracycline (48.57%) (Figure 1a). In case of *Enterococcus* strains, antimicrobial screening tests revealed high susceptibility rates (100%) to penicillin, ampicillin, vancomycin, and fosfomycin. However, high resistance rates have been observed for levofloxacin (84.62%), erythromycin (61.54%), and tetracycline (23.08%) (Figure 1b and Figure 2).

### 2.3. Virulence Factors Expression

#### 2.3.1. Cell Associated Virulence Factors

**Figure 3:**
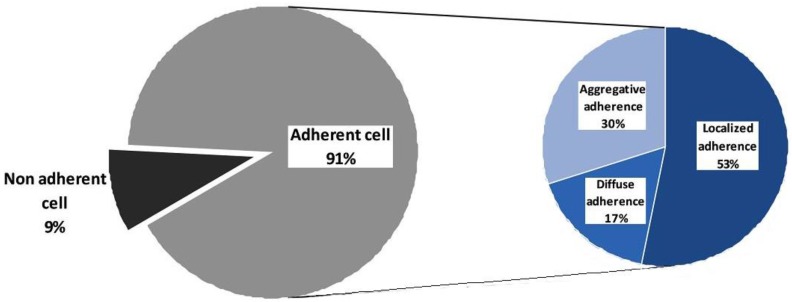
Distribution of the adherence to the cellular substratum among the analyzed uropathogenic strains.

The adherence ability of isolates was evidenced by slime production and in vitro attachment of bacteria to human epithelial-like tumor line (Hep-2) cell monolayer. Slime factor is a hydrophilic exopolysaccharide secreted by some strains, which contributes to bacterial cells’ adherence to inert, abiotic surfaces. Slime factor is an indicator of the resistance and survival capacity in the external environment and can be a virulence factor during the infection of a host organism by opposing the phagocytosis, preventing the access of antimicrobial substances in microbial cells and facilitating adherence to host tissues. Slime production was evident in approximately 50% of the tested strains. *E. coli* strains were the most positive for this virulence factor (44.4%) followed by *Enterococcus* spp. (33%), while *Klebsiella* spp. isolates were negative. The majority of the uropathogenic isolates (90.77%) were able to adhere to Hep-2 cell monolayer in vitro, 65% of the strains exhibiting an adherence index >50%, with localized (53%), aggregative (30%), and diffuse (17%) patterns (Figure 3).

#### 2.3.2. Soluble Virulence Factors

**Figure 4:**
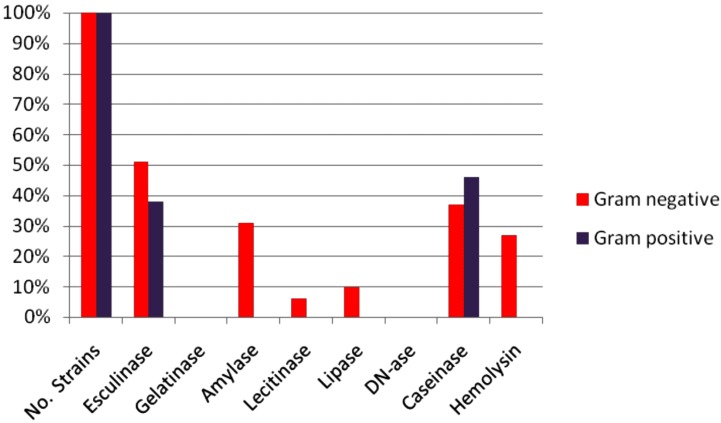
Graphic representation of the profile of soluble virulence factors in the analyzed strains.

The in vitro experiments showed that the uropathogenic isolates were able to produce several soluble metabolic products with potential tissue-damaging effects, exhibiting different profiles represented in the Gram-negative strains by the pore forming toxins (hemolysins (27%), lecithinases (6%) and lipases (10%)), proteases (caseinase (37%)), siderophore-like molecules resulted from the in vitro hydrolysis of esculin (31%) and amylases (31%). The Gram-positive bacteria were positive only for caseinase (46%) and esculin hydrolase (38%) (Figure 2 and Figure 4).

## 3. Discussion

Urinary tract infections are common among elderly patients in residential care facilities, as well as in the hospital settings [17]. With age, men acquire structural and functional abnormalities of the urinary tract that impair normal functioning; the most common is benign prostatic hyperplasia, which can cause urinary tract infection resulting in obstruction and turbulent urine flow. Urinary tract infection in men without indwelling catheters is uncommon among men younger than 60 years old, but the incidence increases substantially after this age. The reported incidence in the community is 0.9 to two cases per 1000 men among those who are younger than 55 years of age and 7.7 cases per 1000 men among those who are 85 years of age or older [18].

The most commonly isolated organism in the present study was *E. coli* (60%), followed by *Enterococcus* spp. (15%) and *Klebsiella* spp. (8.2%). The results are in accordance with previous studies that identified the presence of the same species in 50–60% of these infections [19,20,21,22]. Other enterobacterial species such as *Proteus* spp., *Enterobacter* spp., *Serratia marcescens*, and *Morganella morganii* were recorded, as also revealed by other studies [23]. Gram-positive organisms, such *Enterococcus* spp., are less common overall, but are seen with increasing frequency in healthcare settings and in adults with chronic indwelling catheters [24,25], in which *Pseudomonas* spp., with its intrinsic resistance is also problematic [26,27].

Antibiotic resistance has become a major aspect to be considered in the treatment of community-acquired UTIs [28,29]. Frequently, the diagnosis of UTI is made in the absence of a typical clinical history and signs resulting in overdiagnosis and overtreatment [13]. The importance of culturing prior to instituting primary empirical therapy is exemplified by the fact that, in an area with resistance rates in *E. coli* of 20% to trimethoprim and 10% to fluoroquinolones (representing low figures for many parts of the world today), the risk of treatment failure due to resistance to empirical trimethoprim therapy is 10% [22,30]. Also, the ciprofloxacin resistance in trimethoprim-resistant *E. coli* is not 10% as was anticipated, but 25–40% depending on the reporting country [31]. Cunha et al. (2016) assessed the frequency and susceptibility to antimicrobials of uropathogens isolated from community-acquired urinary tract infections in the city of Natal, Rio Grande do Norte State capital, northeastern Brazil, from 2007 to 2010. They found that most of the isolated uropathogens (*E. coli*, *Klebsiella* spp., and *Staphylococcus* spp.) were susceptible to nitrofurantoin (>92%), excepting *Klebsiella* spp. strains (45%). In a population study in Spain, including both complicated and uncomplicated UTIs in male and female patients, the susceptibility percentages for *E. coli* were low for amoxicillin (41%), trimethoprim–sulfamethoxazole (66%), and ciprofloxacin (77%). Because susceptibility varies with the geographic region and population (nosocomial or community), empiric antibiotic prescription should be dependent on the susceptibility percentages of a specific community over time [32,33]. Regarding antibiotic resistance in Gram-negative bacteria, we observed a high degree of sensitivity to nitrofurantoin and fosfomycin, all strains being susceptible to the last one. The frequent of use of tetracycline, quinolones, and sulfonamides in treatment demonstrated an increasing degree of resistance to these antibiotics.

CTX-M-producing *E. coli* strains isolated from hospital and community sites often exhibit co-resistance to trimethoprim-sulfamethoxazole, tetracycline, gentamicin, tobramycin, and ciprofloxacin [34]. A previous 10-year study from the Calgary Health Region in Calgary, Alberta, Canada, demonstrated that CTX-M-producing *E. coli* is emerging as an important cause of community-onset UTIs [35]. That study showed a substantial increase of CTX-M-15-producers from urines that occurred during the latter part of the study period. It is known that the occurrence of extended-spectrum beta-lactamase (ESBL)-producing *E. coli* in high-risk areas of the hospital—such as intensive care units (ICUs)—has increased significantly [36,37]. Before 2003, most ESBLs strains were *Klebsiella* spp. and were mutants of TEM (from Temoniera, name of the Greek patient) and SHV (sulfhydryl variable) penicillinases. They were often hospital acquired. Recently, the number of CTX-M ESBLs is increasing reported in *E. coli* as well as in *Klebsiella* spp. and many could emerge in community [19]. The former antibiotic therapy with agents, such as cephalosporins, or previous international travel history are recognized as risk factors for the acquisition of these organisms. In our study, 16.47% of the total number of Gram-negative bacteria produced ESBLs, a fact that suggests an important spread of these enzymes in the community, even if these outpatients’ infections are not entirely community acquired and could be correlated with hospital or health care facility visits [38,39,40,41,42,43,44,45,46,47].

Besides their resistance to the current armamentarium of antimicrobial agents, uropathogenic bacterial strains exhibit virulence factors that help the microorganism to overcome host defense mechanisms and colonize or invade the urinary tract [48]. Colonization can lead to the establishment of a quiescent intracellular reservoir in the bladder. Activation of this quiescent intracellular reservoir results in recurrent UTIs [49]. Virulence factors of recognized importance in the pathogenesis of UTIs that have been identified in *E. coli* pathogenic strains, including adhesins (P fimbriae, certain other mannose-resistant adhesins, and type 1 fimbriae), the aerobactin system, hemolysin, K capsule, and resistance to serum killing [50,51]. Fimbriae allow irreversible attachment to the uroepithelial cell membrane via adhesins [52,53]. Adhesion to intestinal cell models has been investigated previously and, in *E. coli* and many other pathogenic species, differences in adherence level and patterns exist [54,55,56,57,58]. Our investigation revealed a high capacity of the uropathogenic isolates to adhere to Hep-2 cell monolayer in vitro, mostly exhibiting a localized adherence pattern.

An essential required step for urinary tract colonization and infection development is microbial production of extracellular polysaccharide polymers or “slime factor” [59]. It acts as the foundation and cement for the formation of microbial biofilms, structures with defined architecture, providing the microorganisms with an excellent protective environment (less susceptible to antibiotics) and favoring the exchange of genetic material (virulence and antibiotic resistance determinants) between cells as well as intercellular communication. Our experimental results showed that the slime factor production, a necessary element in the colonization of urinary tract, was present in 50% of the analyzed strains; *Enterococcus* spp. strains proved to be the most significantly slime forming bacteria (33%). *Klebsiella* spp. isolates were negative for the production of slime factor, while *E. coli* and *Enterobacter* spp. isolates were slime positive. These results demonstrate the potential of the isolated uropathogens to interfere with the treatment of infections, impairing the action of host immune cells and compromising antibiotic efficacy.

Uropathogenic strains are characterized by the expression of a diverse arsenal of microbial products that serves as virulence factors in the pathogenesis of disease by facilitating the spread of bacteria or toxins through tissues [60]. The uropathogenic isolates produced various metabolic soluble virulence factors with possible implications in the pathogenesis process. Differences in the repertoire and expression levels of virulence factors that can affect bacterial growth and persistence within the urinary tract were detected. Thus, more than half of the analyzed uropathogenic strains expressed soluble proteases, which may be used by the bacteria to digest extracellular matrix proteins and polysaccharides. Subsequently, pathogens may invade the host tissue cells and gain access to the intracellular environment [61].

Limiting iron availability is important for host defense against invading bacterial pathogens. Uropathogens have evolved multiple strategies for swiping iron from the host that include the expression of iron acquisition systems that utilize siderophores to scavenge iron from the environment and subsequently concentrate it in the bacterial cytosol. They express a wealth of seemingly redundant iron acquisition systems, including the siderophores salmochelin, yersiniabactin, and aerobactin [62,63,64]. In our study, about 51% of the total number of strains isolated from urinary infection expressed a metabolic feature providing them with the ability to chelate iron, through esculetol, resulting from the esculin hydrolysis.

The resistance–virulence link is complex, considering the diversity of antimicrobial resistance genes, virulence factors, bacterial species and hosts. More in depth molecular studies on the genetic support of antimicrobial resistance and virulence determinants are sorely needed to fully understand the interplay of resistance and virulence genes; whether virulence expression is affected by chromosomal mutations leading to specific resistance (e.g., fluoroquinolone resistance); if both determinants are inserted in the same mobile genetic element, like a conjugative plasmid; and the role of the phylogenetic background of the strain [65,66].

The correlations between resistance spectrums, virulence factors, and recurrence rates are of great clinical value for clinical diagnosis, treatment, and predictive prognosis of recurrent UTIs [65].

## 4. Materials and Methods

### 4.1. Bacterial Cultures and Antimicrobial Susceptibility Testing

Quantitative analyses of urine cultures were performed using the standard calibrated loop method. Urine sample were streaked on Columbia agar with 5% sheep blood and CLED (cysteine-, lactose-, and electrolyte-deficient) agar. After incubation at 37 °C for 24 h, the microorganisms were identified using standard commercial test kits (bioMérieux API). All specimens with bacteriuria of >10^5^ CFU/mL were analyzed to determine the causative pathogens and their antimicrobial susceptibility profile.

Antimicrobial susceptibility testing was performed by disk diffusion method following the guidelines of the Clinical and Laboratory Standards Institute (CLSI) [67]. For Gram-negative bacteria (GNB) were tested following antibiotics (Oxoid Ltd., Basingstoke, UK): amoxicillin–clavulanic acid (AMC 30 μg), cefuroxime (CXM 30 μg), ceftazidime (CAZ 30 μg), norfloxacin (NOR 10 μg), ciprofloxacin (CIP 5 μg), tetracycline (TE 30 μg), trimethoprim–sulfamethoxazole (SXT 25 μg), nitrofurantoin (F 300 μg), fosfomycin (FOT 200 μg), gentamicin (CN 10 μg). For Gram-positive bacteria (GPB) penicillin (P 10 IU), ampicillin (AMP 10 μg), erythromycin (E 15 μg), levofloxacin (LEV 5 μg), tetracycline (TE 30 μg), and nitrofurantoin (F 300 μg) were tested.

### 4.2. Investigation of Cell-Associated and Soluble Virulence Microbial Factors

#### 4.2.1. Adherence Assay

The adherence capacity to the inert substratum of the microbial strains was determined by quantifying the production of slime factor using the microtiter plate method [68]. Microbial suspensions corresponding to 0.5 McFarland density were seeded in nutrient broth, distributed in 96-well plates, then incubated in aerobic conditions at 37 °C for 24 h and 48 h, respectively. After incubation, the plates were washed three times with physiological sterile water for removal of planktonic, not adherent bacteria, fixed with ethanol for 5 min, stained with 1% crystal violet for 20 min, and washed again with tap water. The adherent microbial cells were then resuspended in 33% acetic acid. Intensity of colored suspension, which is directly proportional with the ability of strains to adhere to inert substrate, was measured spectrophotometrically at an optical density of 490 nm (OD490).

The adherence to the cellular substratum was assessed by the Cravioto’s adapted method using Hep-2 line cells cultivated for 24 h at 37 °C in Eagle’s Minimal Essential Medium (MEM) supplemented with antibiotics and 10% fetal bovine serum (Gibco-BRL). The eukaryotic cells were washed three times with sterile phosphate-buffered saline (PBS) and then covered with microbial cell suspensions with a density corresponding to the 0.5 McFarland nephelometric standard prepared in PBS using 18–24 h bacterial cultures. After incubation at 37 °C, for two hours, the eukaryotic cell monolayers were washed three times with PBS, fixed with methanol (5 min) and stained with 10% Giemsa solution for 20 min, washed with tap water, dried at room temperature, and submitted to microscopic examination with the 100× immersion objective, in order to establish the adherence patterns (localized, diffuse, or aggregative) of the microbial isolates and to determine the adherence index (expressed as the ratio between the number of eukaryotic cells exhibiting adhered microbial cells per 100 cells counted on the microscopic field).

#### 4.2.2. Soluble Virulence Factor Production

Agar media incorporating specific enzymatic substrates were used to determine the expression of different metabolic enzymes with potential roles in virulence: hemolysins, amylases, caseinases, gelatinases, esculin hydrolysis, and DNase, as previously reported [69]. Briefly, 18-h microbial culture was spotted onto agar plates with specific enzymatic substrata, i.e., 5% sheep blood (hemolytic activity), 1% starch (amylase activity), 1% casein and 0.4% gelatin (proteolytic activity), 1% esculin (esculinase activity), and 0.2% DNA (DNase production). Enzyme production was detected after 48–72 h of incubation, at 37 °C, by macroscopic observations of specific modifications of the media around the culture spot (hemolysis, precipitation, clearing, or blackening).

## 5. Conclusions

Knowledge of likely organisms and local resistance patterns is crucial in determining appropriate empirical antibiotic treatment in these patients. The increasing prevalence of health care associated infection and emerging antibiotic resistance highlights the importance of obtaining a firm diagnosis, treating with appropriate antibiotics, and avoiding the use of broad-spectrum antibiotics. Antibiotic resistance is increasing and beginning to affect the outcome of empirical antimicrobial therapy of urinary tract infections. An accurate diagnosis of urinary infections is crucial for the choice of the appropriate narrow-spectrum antibiotics.
